# Comparative transcriptome analysis of aerial and subterranean pods development provides insights into seed abortion in peanut

**DOI:** 10.1007/s11103-014-0193-x

**Published:** 2014-05-05

**Authors:** Wei Zhu, Xiaoping Chen, Haifen Li, Fanghe Zhu, Yanbin Hong, Rajeev K. Varshney, Xuanqiang Liang

**Affiliations:** 1 Crops Research Institute, Guangdong Academy of Agricultural Sciences (GAAS), Guangzhou, China; 2 International Crops Research Institute for the Semi-Arid Tropics (ICRISAT), Hyderabad, 502324 India

**Keywords:** Aerial pod, Subterranean pod, Transcriptome, Peanut, Seed abortion, Development

## Abstract

The peanut is a special plant for its aerial flowering but subterranean fructification. The failure of peg penetration into the soil leads to form aerial pod and finally seed abortion. However, the mechanism of seed abortion during aerial pod development remains obscure. Here, a comparative transcriptome analysis between aerial and subterranean pods at different developmental stages was produced using a customized NimbleGen microarray representing 36,158 unigenes. By comparing 4 consecutive time-points, totally 6,203 differentially expressed genes, 4,732 stage-specific expressed genes and 2,401 specific expressed genes only in aerial or subterranean pods were identified in this study. Functional annotation showed their mainly involvement in biosynthesis, metabolism, transcription regulation, transporting, stress response, photosynthesis, signal transduction, cell division, apoptosis, embryonic development, hormone response and light signaling, etc. Emphasis was focused on hormone response, cell apoptosis, embryonic development and light signaling relative genes. These genes might function as potential candidates to provide insights into seed abortion during aerial pod development. Ten candidate genes were validated by Real-time RT-PCR. Additionally, consistent with up-regulation of auxin response relative genes in aerial pods, endogenous IAA content was also significantly increased by HPLC analysis. This study will further provide new molecular insight that auxin and auxin response genes potentially contribute to peanut seed and pod development.

**Electronic supplementary material:**

The online version of this article (doi:10.1007/s11103-014-0193-x) contains supplementary material, which is available to authorized users.

## Introduction

Peanut (*Arachis hypogaea* L.) is an important oilseed and economic crop cultivated in worldwide for providing human nutrition and oil production. Different to other plant, the peanut plant produces flowers aerially, while develops fruit and seeds underground with fascinating gravitropic growth habits (Zhu et al. 2013). In the reproduction cycle, when the fertilization is succeeded after flowering, the ovule-carrying peg (gynophore) starts to form and then down elongation to bury the fertilized ovule into the soil. However, only until the peg carries the ovule into the soil where can the pod normally swell to allow room for the embryo to grow and eventually become subterranean pod (Feng et al. 1995; Moctezuma and Feldman 1999, 2003). The failure of peg penetration into the soil leads to suppression of pod swelling initiation and form aerial pod, finally causing seed abortion and seriously impacting on the peanut production (Chen et al. 2013). For instance, when gynophore penetration into the soil is prevented by any means of a physical barrier but still under a light treatment, the pod will not form normally (Zamski and Ziv 1976; Thompson et al. 1985; Moctezuma 2003). Therefore, it is essential to gain a clearer understanding of these occurring mechanisms during peanut pod development.

Seed formation in peanut is a central stage of pod development. This complex process is initiated by a successful double fertilization that not only results in a diploid embryo and a triploid endosperm, but also triggers development of seed coat by tissue differentiation and cell expansion (Sin et al. 2006; Capron et al. 2012). Accumulating evidence illustrates that seed development is highly coordinated by both endogenous signal and environment stimuli. For instance, several plant hormones have long been known to play a significant role in peanut gynophore elongation and embryo differentiation, such as auxin (Jacobs 1951; Moctezuma and Feldman 1996), the ration of NAA and kinetin (Ziv and Zamskj 1975), ABA (Ziv and Kahana 1988), ethylene (Shlamovitz et al. 1995). In addition, mechanical stimulus and alternation of light and dark conditions also controlled the cessation of embryo differentiation during peg elongation phase, and the resumption of embryo development following quiescence in underground phase (Zamski and Ziv 1976; Stalker and Wynne 1983; Thompson et al. 1985; Shlamovitz et al. 1995; Nigam et al. 1997). At present, despite a comprehensive understanding of physiological and environmental factors that influence seed and pod development, isolation and characterization of candidate genes is of vital importance for improving peanut seed quality and yield.

Over the past decade, with the advent of rapid and high-throughput technology for quantification of the transcriptome (Malone and Oliver 2011), progress on seed development (Guo et al. 2008; Zhang et al. 2012) and tissue expression (Payton et al. 2009; Wang et al. 2012) in peanut (Haegeman et al. 2009; Tirumalaraju et al. 2011; Chen et al. 2012) has been studied intensely using DNA microarrays or RNA sequencing. For instance, they are explored to investigate how the transcriptome is deployed in aerial and subterranean pods (Chen et al. 2013), and how gene expression varies in response to disease infection (Guo et al. 2008; Wang et al. 2012). Furthermore, in our previous studies (Chen et al. 2013; Zhu et al. 2013), both RNA-seq and proteomics analysis shed light on the potential candidate genes and proteins that regulated aerial and subterranean pods development. These studies not only revealed that the embryo of the aerial pod ceased growth at early stages and finally aborted, but also underlined two senescence associated genes and one late embryogenesis-abundant gene as candidates to embryo abortion of aerial pod; additionally, proteins involved in lignin synthesis and ubiquitin proteasome system might regulate pods swelling to allow room for embryo development.

However, in previous RNA-seq analysis, limited knowledge is available in candidate genes that potentially contribute to seed abortion during aerial pods development. Little is known about stage-specific genes expression alternation during aerial and subterranean pods development due to pooling many samples from aerial and subterranean pods for one aerial library, and two subterranean libraries, respectively. Recently, many studies proved that microarrays remained useful and accurate tools for measuring gene expression alternation across development stages (Bloom et al. 2009; Willenbrock et al. 2009; Bradford et al. 2010), which not only achieved mature and stable analytic strategies, but also developed appropriate standards for this tools (Stears et al. 2003; Malone and Oliver 2011). Therefore, both DNA microarrays and RNA sequencing could complement with each other to profile the transcriptome for addressing problems during peanut aerial and subterranean pods development. In this study, to better understand the mechanism of seed abortion and pod development, we compared the transcriptome profiles of peanut aerial and subterranean pods at different developmental stages by microarrays approaches combined with previous RNA-Seq and proteomics analysis. The objectives of the present study were to: (1) compare the differentially expression of genes between developing aerial and subterranean pods; (2) identify potential candidate genes related to seed abortion; and (3) highlight stage-specific genes expression alternation during aerial and subterranean pods development.

## Materials and methods

### Plant materials and treatment

A peanut cultivar, ‘Yueyou-7’, was provided by Crops Research Institute, Guangdong Academy of Agricultural Sciences (GAAS, China). We identified selfed flowers with colored plastic thread, and marked elongating aerial pegs by tying with colored tags at the eighth day after flowering (DAF). After these treatments, one-third of marked pegs were artificially covered with soil, while the other two-third were put thick plastic membrane to prevent them from penetrating into the soil. Both of them were of the same age and grown in experimental flowerpot with normal management. We collected aerial pods and subterranean pods at 1, 2, 4, 8 days after marked (DAM), corresponding to 9, 10, 12, 16 DAF. In order to get the same parts of the pods, all important components such as the ovules and meristem from aerial and subterranean pods were excised. Especially on the early development stages, the same length (about 10 mm) from the tips of aerial and subterranean pods was excised. At the 4 and 8 DAM, aerial pods were excised around 10 mm from the apex, while subterranean pods were collected the swelling part for total RNA isolation. The materials were collected and immediately frozen in liquid nitrogen, and then stored in a freezer at −80 °C.

### Fixation, sectioning and staining

Material was fixed in FAA (50 % alcohol:acetic acid:formaldehyde solution = 89:6:5) at room temperature. The samples were then vacuum infiltrated to remove trapped air. Samples were washed by 50 % alcohol, dehydrated using an ethyl alcohol series, cleared in xylene and embedded in paraffin wax. The specimens were sectioned to a thickness of 8 μm. Sections were stained with toluidine blue, observed and photographed using a Leica DMLB light microscope (Leica Microsystems GmbH, Wetzlar, Germany).

### RNA extraction and microarrays procedure

Total RNA for microarray and quantitative real-time RT-PCR analysis were isolated from the same aerial and subterranean pods samples using a modified CTAB-based protocol (Chang et al. 1993) with high salt and further purified with the RNeasy Plant Mini Kit (Qiagen, Shanghai, China). A NanoDrop ND-1000 Spectrophotometer (Nanodrop Technologies, Wilmington, DE) and agarose gel electrophoresis were used to test RNA quality and quantity. Gene expression profiles were generated using a high-density peanut microarray with pooling three biological replicates together for each development stage. Each microarray used a customized NimbleGen oligonucleotide microarray (4X 72 k) representing 36,158 unigenes.

The microarray hybridization procedure in this study was performed same as our lab Chen’ methods (Chen et al. 2012). Probes on the microarray ranging from 60 to 70 mer were synthesized by Sigma-Aldrich (Saint Louis, MO, USA) and then spotted to Corning ultraGAPs glass slides with three replications of each oligonucleotide at different locations on the slide to accommodate bioinformatics statistic analysis. Microarray procedure was performed according to the methods described previously by our lab Wang et al. (2012). Double-stranded cDNA synthesis, cleaned, fluorescently labeling, microarrays hybridization, washing and scanning were conducted at CapitalBio Corporation (Beijing, China) using Roche (Shanghai, China) NimbleGen Systems.

### Data analysis and functional annotation

All microarrays were scanned with a LuxScan 10 KA scanner using LUXSCAN 4.0 software (CapitalBio, Beijing, China) to generate the raw data files. Fluorescence data were processed with SpotData software to quantify signal at CapitalBio Corporation as described previously (Graubert et al. 2007). For statistical analysis, the data normalization was performed by rank-consistency-filtering with Lowess intensity normalization method based on a robust multichip analysis (RMA, CapitalBio). And expression ratios were collected only on those spots with signal intensity (Cy3) ≥400 in at least one dye channel on the microarray slides after subtraction of the background in all experiments. Statistical analysis involved unpaired *t* test with using of GeneSpringGX11 (Agilent Technologies). The Benjamini-Hochberg FDR method was used to obtain corrected *P* values (false discovery rate, FDR) for multiple testing (*p* ≤ 0.05). The microarray analysis was employed to measure global gene expression in aerial and subterranean pods across four different developmental days. Comparative transcriptomics analysis was conducted between aerial pods and subterranean pods at the same developmental days. And we set the RNA sample from aerial pods as the reference. The intensity values of each sample were further transformed on log_2_-scale and used for performing differential expression analysis. The probe sets had a *P* value < 0.01 and >twofold changes in at least one of the comparisons were considered as differentially expressed genes (DEGs) for further analysis. To show the alternation of each DEGs during the whole development stages, their fold-change data were imported into MultiExperiment Viewer (MeV v4.8, http://www.tm4.org/mev/) for hierarchical clustering analysis with the average linkage method. Venn diagram was also constructed with the Venny tool (Oliveros 2007) to show the stage-specific expressed genes in each comparison and the overlaps between four binary comparison groups. The normalized data, Gene ID, gene sequence, Probes ID and probes sequence from peanut aerial and subterranean pods development microarray expression analysis were listed in Supplemental Table 7, 8 and 9.

The gene annotation was performed according to the method described by Shi et al. (2006). Descriptions of each DEGs were BLAST searched against the NCBI protein database (http://www.ncbi.nlm.nih.gov) and UniProt database (http://www.uniprot.org) for retrieval of updated annotation of homologous proteins with a cutoff of 1E−5. The gene annotation was based on the similarity (>80 % identity) of the homologous proteins and the evolutionary relationship between species (mainly the legume family and *Arabidopsis thaliana*). The DEGs of each comparison and their annotation were listed in Supplemental Table 3. Furthermore, the UniProtKB accession numbers assigned to the DEGs were submitted to gene ontology (GO) terms using MAS (molecule function annotation system, http://bioinfo.capitalbio.com/mas) to organize genes into hierarchical categories on the basis of biological process and molecular function. GO terms with false discovery rate (FDR) corrected *P* values < 0.05 were considered statistically significant.

### Real-time PCR quantification

First-strand cDNA was synthesized from 1 μg total RNA using the ReverTra Ace-α-First Strand cDNA Synthesis kit (TOYOBO). Gene-specific primers were designed with the Primer Premier 5.0 (PREMIER Biosoft International, USA). Quantitative real-time RT-PCR was performed with Realtime PCR Master Mix (TOYOBO) and a LightCycler 480 instrument (Roche) equipped with LightCycler Software Version 1.5 (Roche) based on the manufacturer’s instructions. The *actin gene* was amplified along with the target gene as reference to normalize expression between different samples. All assays for a target gene were performed in triplicate synchronously under identical conditions.

### Plant hormone extraction and measurement

The method for the extraction and purification of endogenous plant hormones GA_3_ and IAA were modified from those described by Kojima (1995) and Yang et al. (2000). One gram plant tissue were ground into fine powder in liquid nitrogen, then added 5 ml 80 % (v/v) methanol containing 10 mg/l butylated hydroxytoluene (BHT) as an antioxidant and transferred this mixture into a conical tube. The methanolic extracts were kept for continuous stirring at 4 °C in the dark for about 12 h. Then centrifuged with 5,000 rpm for 30 min at 4 °C. This procedure was repeated for twice and put the supernatant together, then concentrated to a water residue in vacuum at 35 °C by rotatory evaporation. The volume was adjusted to 2 ml with distilled water and added two volumes of cold chloroform to wash them. Centrifuged at 5,000 rpm for 5 min at 4 °C and adjusted the aqueous phase to pH 2.7 with HCl. The ethyl acetate was layered to the aqueous phase and the two-phase system was gently stirred for 3 min. Repeated for thrice and put the ethyl acetate phase together. The combined ethyl acetate phases were reduced to dryness in vacuum at 35 °C by rotatory evaporation. The solid residue was dissolved in 500 μl 100 % (v/v) methanol, and further filtered to measure GA_3_ and IAA by high performance liquid chromatograph (HPLC) according to Ross’ methods (Ross et al. 2004). All the measurements were performed with three biological replicates together for each development stage.

## Results

### Anatomical analysis of aerial pod

**Fig. 1:**
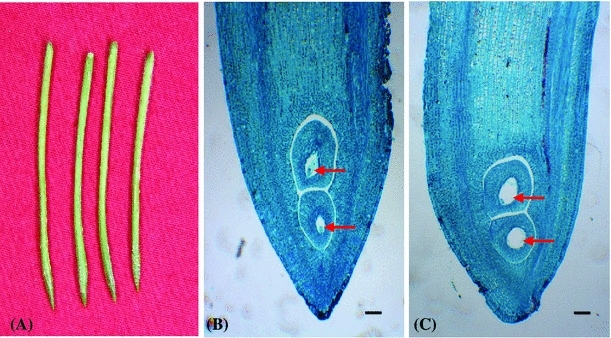
Anatomical analysis of the peanut aerial pod. The embryo of aerial pod was abortion at the late development stage. **a** the aerial pod; **b** Longitudinal section of early aerial pod; **c** Longitudinal section of old aerial pod. Embryos are indicated with *red arrows*. *Bars* = 200 μm

It is still obscure why the aerial pod always remains small pod and can’t develop normally. To better understand the cellular structure of aerial pod, we conducted anatomical analysis by toluidine blue staining of tissue sections. The results revealed that the aerial pod was a special reproductive organ, which showed unique anatomical and morphological characteristics (Fig. 1a–c). The shape of aerial pod looked like a needle, and the fertilized ovary was located at nearly 3–5 mm after the tip (Fig. 1a, b). While at the late development stage, the embryo was abortion due to the failure of aerial pod penetration into the soil (Fig. 1c).

### Differential expression in peanut pod development

**Fig. 2:**
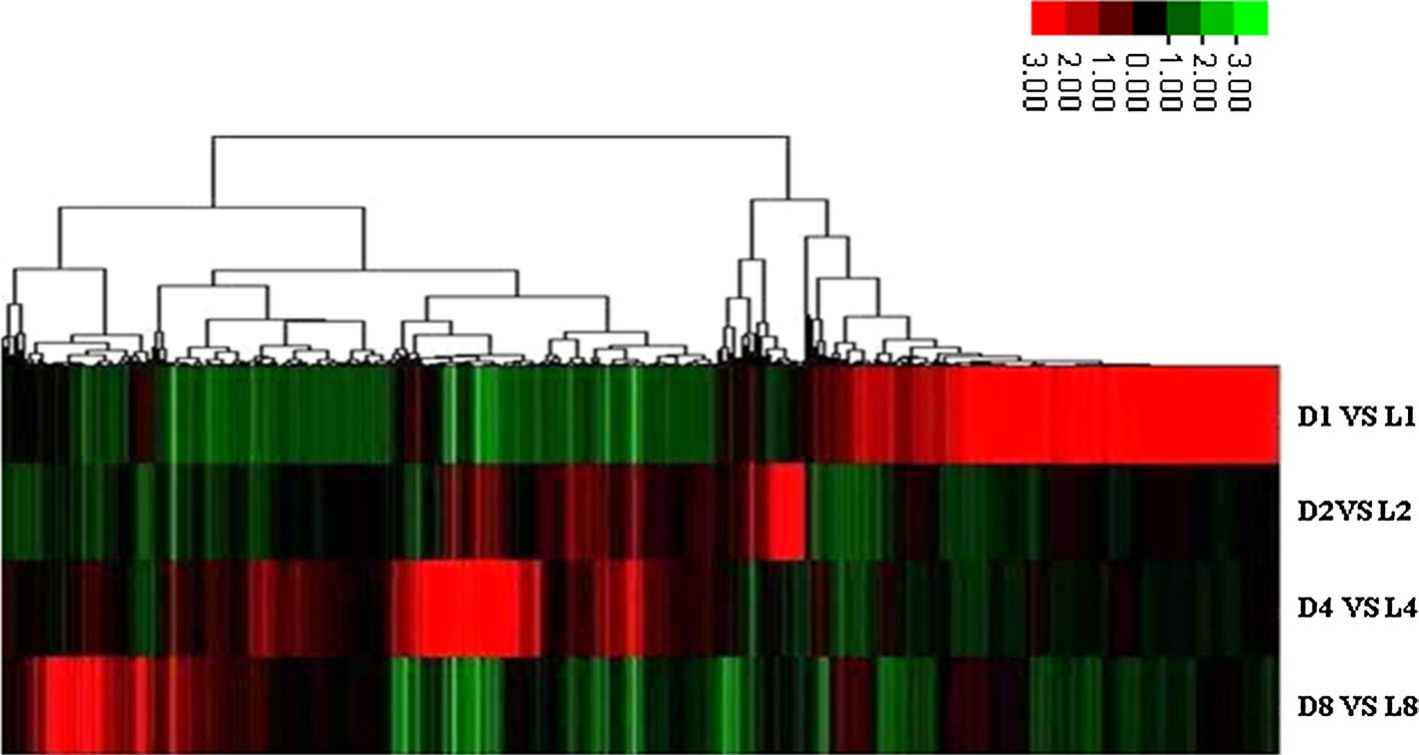
The result of clustering analysis on the differentially expressed genes of subterranean pods versus aerial pods at the different development days. Probe sets with *P* < 0.01 and fold changes (FC) >2 in at least one of the comparison are included. The columns are sorted by hierarchical clustering using the average linkage methods. The ratios are shown in a *red-green color* scale, where red indicates up-regulation and *green* indicates down-regulation. Each row represents a sample of subterranean pods versus aerial pods obtained from three biological replicates and each column represents a differentially expressed probe set. D1 versus L1, D2 versus L2, D4 versus L4 and D8 versus L8: the comparison of subterranean pods versus aerial pods at 1, 2, 4 and 8 days after marked

As shown in the previous study, the seed in aerial pods aborted at the 6 days after marked (DAM) (Chen et al. 2013). To identify differentially expressed genes (DEGs) relating to seed abortion during peanut aerial and subterranean pods development, we used a customized NimblerGen onligonucleotide microarrays at the 1, 2, 4, 8 DAM. Results showed that a total of 18,366 genes, accounting for 50.79 % of all transcripts in the microarrays, were expressed in all the eight samples and 7,835 genes (21.67 %) were not expressed in all the eight samples. In addition, many specific expressed genes which detected only in aerial or subterranean pods across four time-points were identified. Among them, 1,698 genes were detected only in aerial pods (Supplemental Table 1), while 703 genes only in subterranean pods (Supplemental Table 2). We set the sample of aerial pods as control, and totally 6,203 genes were differentially expressed in aerial and subterranean pods development across 4 consecutive time-points (Supplemental Table 3). All of these DEGs showed up-regulation or down-regulation with at least twofold changes. Clustering analysis showed that the expression profiles of DEGs varied significantly in developing peanut aerial and subterranean pods at different DAM (Fig. 2). Based on the expression pattern of subterranean pods versus aerial pods at 1, 2, 4 and 8 DAM, we classified these DEGs into the following 4 major groups, with a few exceptions: group I, a large group of genes whose expression significantly increased at 1 DAM but down-regulated at other DAM; group II, a group of genes whose expression significantly increased at 2 DAM but down-regulated at other DAM; group III, a large group of genes whose expression significantly increased at both 2 and 4 DAM, together with a large group of genes whose expression significantly increased at both 4 and 8 DAM; group IV, a group of genes whose expression significantly increased at 8 DAM but down-regulated at other DAM. The clustering result indicated that seed aborted in aerial pods was a complex process and coordinated by a large group of DEGs at different development stages.

### Comparative analysis of aerial and subterranean pods development

**Fig. 3:**
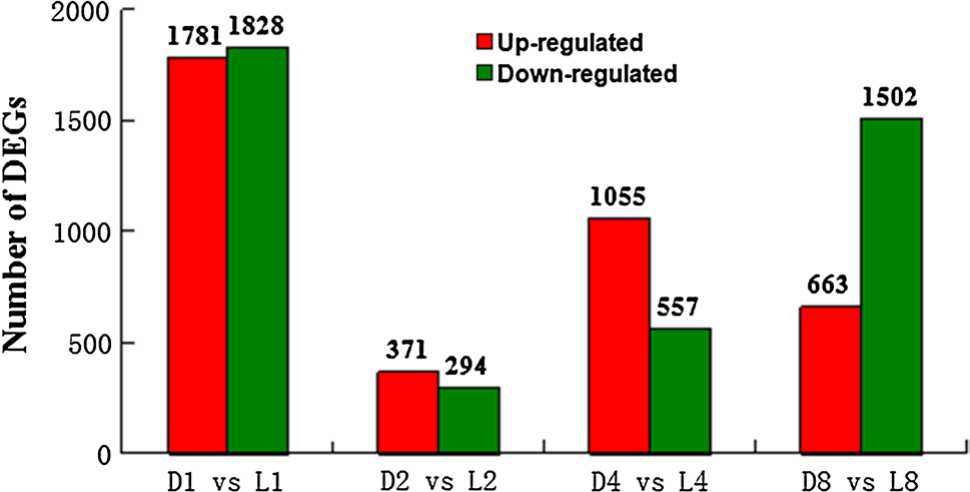
Comparative analysis of differentially expressed genes (DEGs) between aerial and subterranean pods at 1, 2, 4, 8 days after marked. The number of up-regulated and down-regulated genes between aerial and subterranean pods at different DAM are indicated. D1, D2, D4 and D8: the development of subterranean pods after marked at 1, 2, 4 and 8 days; L1, L2, L4 and L8: the development of aerial pods after marked at 1, 2, 4 and 8days

**Fig. 4:**
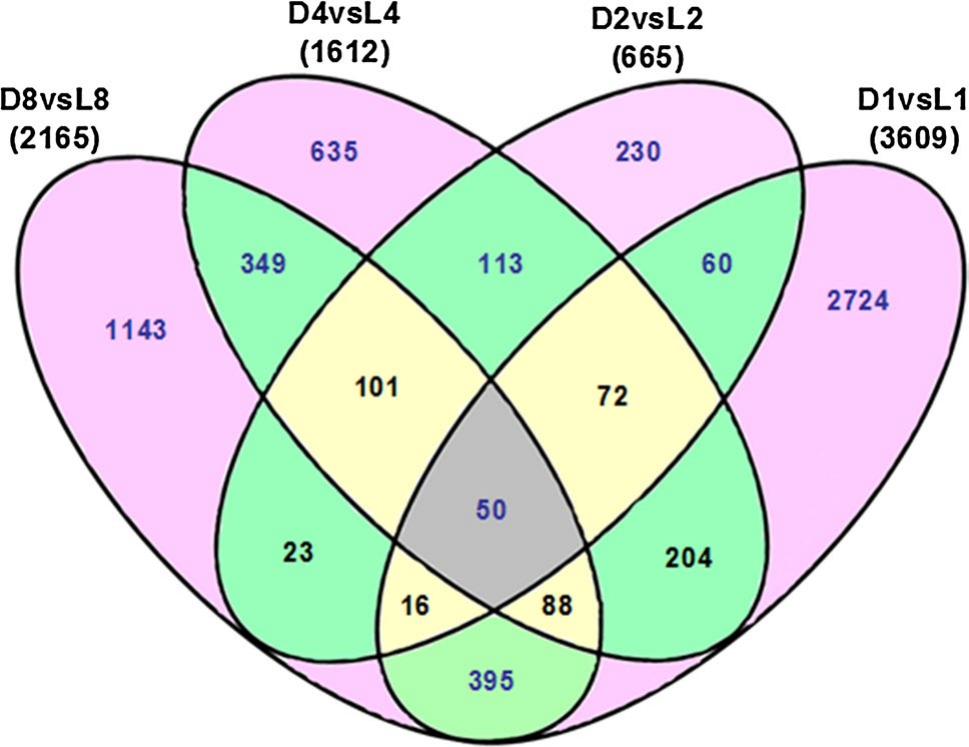
Venn diagram depicting the number of differentially expressed genes between aerial and subterranean pods development at 1, 2, 4 and 8 days after marked in each comparison and the overlaps between four binary comparison groups. For each developmental stage, the total number of differentially expressed genes and the number of stage-specific expressed genes are indicated. D1 versus L1, D2 versus L2, D4 versus L4 and D8 versus L8: the comparison of subterranean pods versus aerial pods at 1, 2, 4 and 8 days after marked

To investigate expression alternation of stage-specific genes and seed abortion candidate genes during aerial and subterranean pods development, we conducted comparative analysis of the transcriptome profiles between aerial and subterranean pods development at 1, 2, 4, 8 DAM. As shown in Fig. 3, comparing subterranean pods with aerial pods, considerable DEGs were identified at 1, 2, 4, 8 DAM. Totally 3,609 differentially expressed genes were detected at 1 DAM (D1 vs L1), including 1,781 up-regulated and 1,828 down-regulated genes. Similarly, 665 DEGs (including 371 up-regulated and 294 down-regulated genes), 1,612 DEGs (including 1,055 up-regulated and 557 down-regulated genes), 2,165 DEGs (including 663 up-regulated and 1,502 down-regulated genes) were identified at 2, 4, 8 DAM (D2 vs. L2, D4 vs. L4, D8 vs. L8), respectively (Supplemental Table 3). These results indicated that dramatical changes in transcriptome profiles of peanut pod development began to occur at the 1 DAM when aerial pegs penetrated into the soil. In addition, many stage-specific DEGs were also analyzed across 4 consecutive time-points using a Venn diagram (Fig. 4). Approximately 2,724, 230, 635 and 1,143 stage-specific DEGs were identified at 1, 2, 4, 8 DAM, respectively. And 50 DEGs were shared among all 4 consecutive time-points. These DEGs could be contributed to seed and pod development under disparate conditions, especially light conditions.

### Gene ontology enrichment analysis for differentially expressed genes

**Fig. 5:**
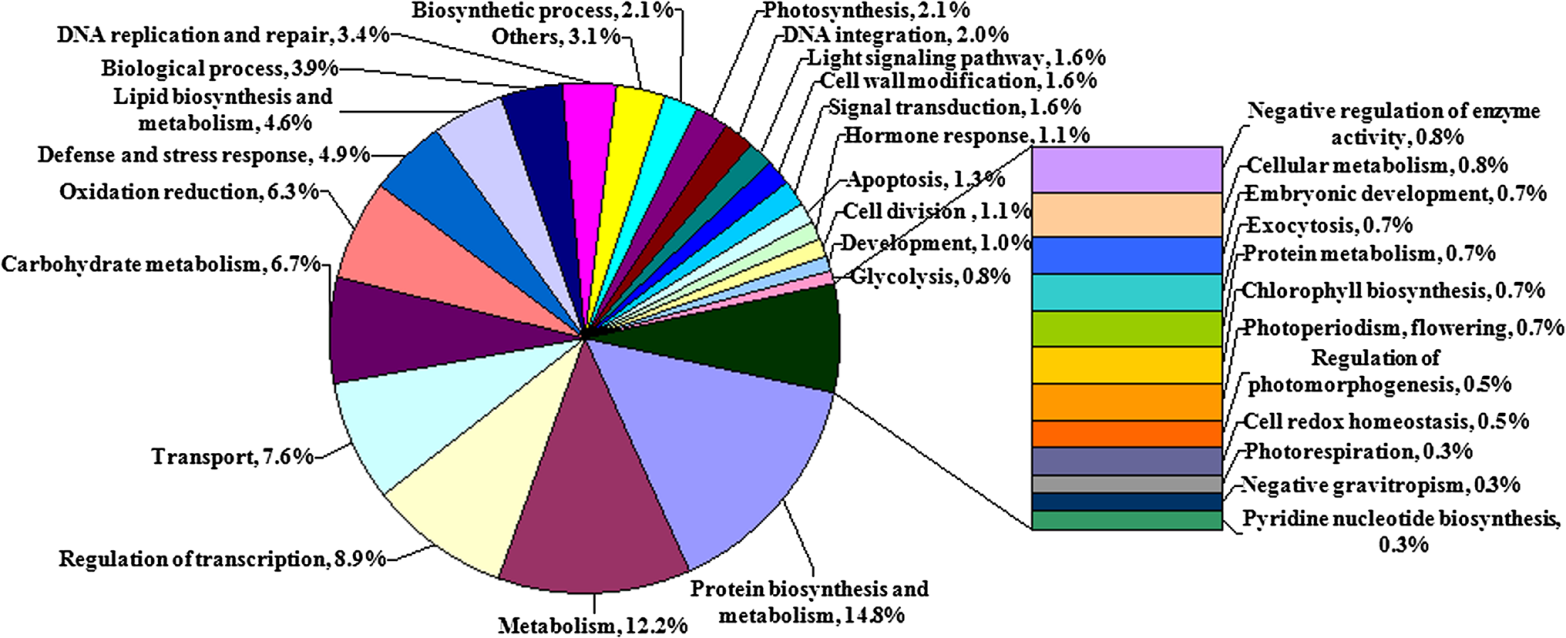
Statistics of differentially expressed genes assigned to GO functional categories based on biological process. Some genes are assigned to more than one GO functional category for participating in multiple biological processes. The percentages for GO terms are calculated by the number of DEGs in one GO term dividing to the total number of DEGs in all GO term

To identify biological process of differentially expressed genes (DEGs), gene ontology (GO) analysis was conducted using Molecule Annotation System (MAS, http://bioinfo.capitalbio.com/mas3). The GO analysis obtained using the annotation procedure through homology analysis to generate a concise functional annotation. As shown in Fig. 5, the known DEGs were mainly classified into 29 functional categories and involved in 34 biological processes. The results showed that these DEGs mainly distributed in plasma membranes and nucleus after genes expression, and participated in the biological process of biosynthesis (2.1 %), metabolism (12.2 %), regulation of transcription (8.9 %), transporting (7.6 %), stress response (4.9 %), cell division and differentiation (1.1 %), cell apoptosis (1.3 %), hormone response (1.1 %), embryonic development (0.7 %), photomorphogenesis (0.5 %), photoperiodism (0.7 %), photosynthesis (2.1 %), lignin synthesis (1.6 %), and so on. All these results indicated the biological process of DEGs varying in a broad range. Through comparative analysis, the two most abundant sub-classes were biosynthesis processes and metabolic processes. Six other subclasses, including regulation of transcription, transport, oxidation and reduction processes, defense and stress response were also enriched. However, there were several important subcategories for pod development which were represented by genes for hormone response, signal transduction and embryonic development.

Furthermore, to determine the biological significance of the differentially expressed genes, GO terms enrichment analysis of the total DEGs was also performed (*P* ≤ 0.05) using the agriGO tools (http://bioinfo.cau.edu.cn/agriGO/). As shown in Supplemental Table 4, the biological processes of response to stimulus (GO:0050896), response to stress (GO:0006950), response to hormone stimulus (GO:0009725) and developmental process (GO:0032502) were enriched GO terms, indicating that hormone and environment stimuli played a vital role in peanut pods development. In the tree traversing of enriched GO terms such as response to hormone stimulus (GO:0009725), 19 DEGs were involved in response to auxin stimulus (GO:0009733); 8 DEGs were involved in response to gibberellin stimulus (GO:0009739); 7 DEGs were related to abscisic acid stimulus (GO:0009737); 5 DEGs were associated to ethylene stimulus (GO:0009723); 7 DEGs were participated in hormone-mediated signaling pathway (GO:0009755). Moreover, GO terms enrichment analysis of the stage-specific DEGs at 1, 2, 4 and 8 DAM were conducted. During aerial and subterranean pods development, the significant GO for stage-specific expressed genes at 1 DAM were mainly classified into the following categories: ion binding (GO:0005506), transferase activity(GO:0016740), catalytic activity (GO:0003824), DNA binding (GO:0003677), oxidoreductase activity (GO:0016491), hydrolase activity (GO:0016787), enzyme activity (GO:0004857), transporter activity (GO:0005215), ATP binding (GO:0005524) and peptidase activity (GO:0004176). Similarly, stage-specific expressed genes at 2 and 8 DAM were significantly enriched with ion binding (GO:0005506). The enrichment GO term for stage-specific expressed genes at 4 DAM mainly belonged to ion binding (GO:0005506), transferase activity(GO:0016740), catalytic activity (GO:0003824), enzyme activity (GO:0004857), ATP binding (GO:0005524) and cofactor binding (GO:0048037).

### Candidate genes related to seed abortion during aerial pods development

Many physiological studies revealed that seed development in peanut was highly coordinated by plant hormones, and also mainly controlled by the alternation of light and dark conditions. Based on GO analysis, some differentially expressed genes participated in biological process of hormone response, cell apoptosis, embryonic development and light signaling. We speculated that these differentially expressed genes might function as candidate genes to provide insights into seed abortion during aerial pods development. These potential candidate genes contained 39 hormone response relative genes, 16 cell apoptosis relative genes, 17 embryonic development relative genes and 10 light signaling relative genes, respectively (Table 1). In the identified hormone relative genes, many shared homology with genes coding for proteins well known to be involved in cell apoptosis, development and light signaling pathways. It indicated that hormone relative genes were in a central position of signaling pathway to regulate seed abortion during aerial pods development. Especially, 19 auxin-related genes, accounting for 48.7 % of all hormone relative genes, were significant differentially expressed during seed and pod development, suggesting that auxin response factor and auxin-induced protein might be involved in seed abortion. However, these genes that might lead to seed abortion need to be confirmed in further functional studies.

**Table 1:** The annotation of candidate genes related to seed abortion during aerial pods development

Gene ID	Uniprot NO.	Species	Protein name	E value
Hormone response relative genes				
AHTC1008761	Q2HRH3	*Medicago truncatula*	Gibberellin regulated protein	1.00E−46
AHTC1031559	A9P6A4	*Medicago truncatula*	Ethylene-responsive transcription factor 1A	8.00E−21
AHTC1009678	O04280	*Phaseolus vulgaris*	Gibberellin 20-oxidase	1.00E−141
AHTC1027127	B9R824	*Ricinus communis*	Auxin-induced protein 5NG4	4.00E−39
AHTC1021447	Q8GV76	*Medicago truncatula*	Auxin efflux carrier protein	3.00E−72
AHTC1035475	A2Q374	*Medicago truncatula*	Gibberellin regulated protein	2.00E−41
AHTC1012926	B9SHD1	*Ricinus communis*	BRASSINOSTEROID INSENSITIVE 1-associated receptor kinase 1	4.00E−66
AHTC1013072	Q76FZ8	*Pisum sativum*	Brassinosteroid receptor	2.00E−75
AHTC1007964	B9R7Q4	*Ricinus communis*	BRASSINAZOLE-RESISTANT 1 protein	1.00E−37
AHTC1015327	B9RWA6	*Ricinus communis*	Gibberellin receptor GID1	2.00E−31
AHTC1026621	Q6L8U0	*Cucumis sativu*	Auxin response factor 4	2.00E−33
AHTC1021732	B9N158	*Populus trichocarpa*	Auxin efflux carrier component	1.00E−58
AHTC1028384	Q8GV76	*Medicago truncatula*	Auxin efflux carrier component	2.00E−83
AHTC1008463	B9S5C3	*Ricinus communis*	Ethylene-overproduction protein	6.00E−46
AHTC1013682	Q9ATR0	*Pisum sativum*	Brassinosteroid biosynthetic protein LKB	2.00E−92
AHTC1004450	Q05G09	*Lupinus albus*	Auxin efflux carrier	7.00E−96
AHTC1016323	C6ZJZ5	*Glycine max*	Auxin efflux carrier protein 2	2.00E−76
AHTC1003763	B9I0L4	*Populus trichocarpa*	Auxin efflux carrier family protein	3.00E−124
AHTC1020717	Q05680	*Glycine max*	Auxin-responsive GH3 product	1.00E−54
AHTC1000327	Q45W71	*Arachis hypogaea*	Auxin-repressed protein	2.00E−43
AHTC1017738	Q8S4Q2	*Arachis ipaensis*	Ethylene-responsive transciptional coactivator-like protein	6.00E−58
AHTC1014115	A5HSG1	*Arachis ipaensis*	Ethylene-responsive transcription factor	3.00E−36
AHTC1034938	Q76FZ8	*Pisum sativum*	Brassinosteroid receptor	5.00E−26
AHTC1000612	Q4W8C3	*Phaseolus angularis*	Gibberellin 2-oxidase	3.00E−125
AHTC1010480	Q9FNV7	*Robinia pseudoacacia*	Auxin-repressed protein	7.00E−39
AHTC1026648	B0L633	*Cicer arietinum*	GA-like protein	5.00E−19
AHTC1008511	B9SWW7	*Ricinus communis*	Auxin response factor GTPase activator	1.00E−73
AHTC1023667	A9QNE7	*Solanum lycopersicum*	ABA 8’-hydroxylase	7.00E−36
AHTC1005584	B6VB01	*Arachis hypogaea*	Auxin binding protein 1	4.00E−99
AHTC1003543	Q94F62	*Arabidopsis thaliana*	BRASSINOSTEROID INSENSITIVE 1-associated receptor kinase 1	2.00E−12
AHTC1006456	Q0GXX3	*Medicago truncatula*	Auxin conjugate hydrolase	0
AHTC1004284	Q8S4Q2	*Ammopiptanthus mongolicus*	Ethylene-responsive transciptional coactivator-like protein	6.00E−28
AHTC1025214	B2BA73	*Pisum sativum*	Gibberellin 3-oxidase	1.00E−28
AHTC1009513	B9STH7	*Ricinus communis*	Auxin-induced protein 5NG4	4.00E−64
AHTC1032952	Q45W71	*Arachis hypogaea*	Auxin-repressed protein	2.00E−18
AHTC1034412	Q8W3P8	*Phaseolus angularis*	ABA-glucosyltransferase	6.00E−60
AHTC1022224	P33081	*Glycine max*	Auxin-induced protein 15A	5.00E−21
AHTC1019083	P33079	*Glycine max*	Auxin-induced protein 10A5	4.00E−31
AHTC1030208	P33080	*Glycine max*	Auxin-induced protein X10A	8.00E−34
Cell apoptosis relative genes				
AHTC1006425	B9RDP2	*Ricinus communis*	Dead box ATP-dependent RNA helicase	2.00E−72
AHTC1016793	B9T0X5	*Ricinus communis*	Dead box ATP-dependent RNA helicase	6.00E−63
AHTC1010861	B9RWT5	*Ricinus communis*	Dead box ATP-dependent RNA helicase	7.00E−85
AHTC1020385	Q0H950	*Glycine max*	Lethal leaf spot 1-like protein	2.00E−95
AHTC1004442	A3QRM3	*Glycine max*	Senescence-associated nodulin 1A	6.00E−76
AHTC1008715	Q0H950	*Glycine max*	Lethal leaf spot 1-like protein	1.00E−62
AHTC1010962	D3G9M3	*Glycine max*	Vascular associated death 1	2.00E−52
AHTC1008318	B5TV63	*Camellia sinensis*	Senescence-related protein	7.00E−62
AHTC1004846	B9RDP2	*Ricinus communis*	Dead box ATP-dependent RNA helicase	4.00E−138
AHTC1027355	Q2HVE0	*Medicago truncatula*	Leucine-rich repeat	2.00E−17
AHTC1025672	Q2YE88	*Glycine max*	NB-LRR type disease resistance protein Rps1-k-1	5.00E−15
AHTC1014344	Q2YE88	*Glycine max*	NB-LRR type disease resistance protein Rps1-k-1	6.00E−35
AHTC1028456	Q84ZU8	Glycine max	R 10 protein	3.00E−53
AHTC1033023	Q84ZU5	Glycine max	R 8 protein	8.00E−31
AHTC1035719	Q8W2C0	Glycine max	candidate resistance protein KR1	4.00E−21
AHTC1003543	Q94F62	*Arabidopsis thaliana*	BRASSINOSTEROID INSENSITIVE 1-associated receptor kinase 1	2.00E−12
Embryonic development relative genes				
AHTC1032586	B4UW62	*Arachis hypogaea*	Embryo-abundant protein EMB	2.00E−22
AHTC1010653	Q9SWB3	*Glycine max*	Seed maturation protein PM39	2.00E−22
AHTC1001743	Q39871	*Glycine max*	Late embryongenesis abundant protein	2.00E−56
AHTC1000013	O49817	*Cicer arietinum*	Late embryogenesis abundant protein 2	1.00E−36
AHTC1001476	Q39801	*Glycine max*	51 kDa seed maturation protein	6.00E−26
AHTC1014629	Q39871	*Glycine max*	Late embryongenesis abundant protein	1.00E−58
AHTC1001477	Q39801	*Glycine max*	51 kDa seed maturation protein	7.00E−49
AHTC1014265	O49817	*Cicer arietinum*	Late embryogenesis abundant protein 2	7.00E−19
AHTC1014482	O49817	*Cicer arietinum*	Late embryogenesis abundant protein 2	3.00E−38
AHTC1000135	O49817	*Cicer arietinum*	Late embryogenesis abundant protein 2	3.00E−39
AHTC1005600	Q2XSI1	*Glycine latifolia*	Seed maturation protein	1.00E−23
AHTC1006589	Q9ZTZ3	*Glycine max*	24 kDa seed maturation protein	3.00E−54
AHTC1011324	Q9SWS4	*Glycine max*	Ripening related protein	1.00E−43
AHTC1003523	Q9SWS4	*Glycine max*	Ripening related protein	1.00E−36
AHTC1014148	O49817	*Cicer arietinum*	Late embryogenesis abundant protein 2	4.00E−7
AHTC1011166	Q9SYM4	*Arabidopsis thaliana*	alpha-trehalose-phosphate synthase	2.00E−136
AHTC1022204	O49552	*Arabidopsis thaliana*	DNA damage-binding protein 1b	5.00E−30
Light signaling relative genes				
AHTC1014391	Q8LEA8	*Arabidopsis thaliana*	Phytochrome A-associated F-box protein	7.00E−44
AHTC1029353	B9MST1	*Glycine max*	Circadian clock-associated FKF1	2.00E−92
AHTC1003429	B9MST1	*Glycine max*	Circadian clock-associated FKF1	0
AHTC1021859	B9MST1	*Glycine max*	Circadian clock-associated FKF1	5.00E−49
AHTC1000126	Q850G4	*Arachis hypogaea*	Putative early light induced protein	6.00E−98
AHTC1000086	Q850G4	*Arachis hypogaea*	Putative early light induced protein	9.00E−22
AHTC1024152	Q8GWZ0	*Arabidopsis thaliana*	uncharacterized protein	3.00E−06
AHTC1013035	Q8GWZ0	*Arabidopsis thaliana*	uncharacterized protein	2.00E−26
AHTC1022204	O49552	*Arabidopsis thaliana*	DNA damage-binding protein 1b	5.00E−30
AHTC1024768	Q5XEU1	*Arabidopsis thaliana*	At2g21070	5.00E−16

The eighty-two candidate genes identified in this study are shown. Gene ID is provided on the left side of the table. Based on the functional annotation and GO analysis as described in the section of materials and methods, they are mainly hormone response, cell apoptosis, embryonic development and light signaling relative genes. Their hits of Uniprot accession number, species, protein name and E value are shown in the table

### Validation of microarrays data by real-time RT-PCR

**Fig. 6:**
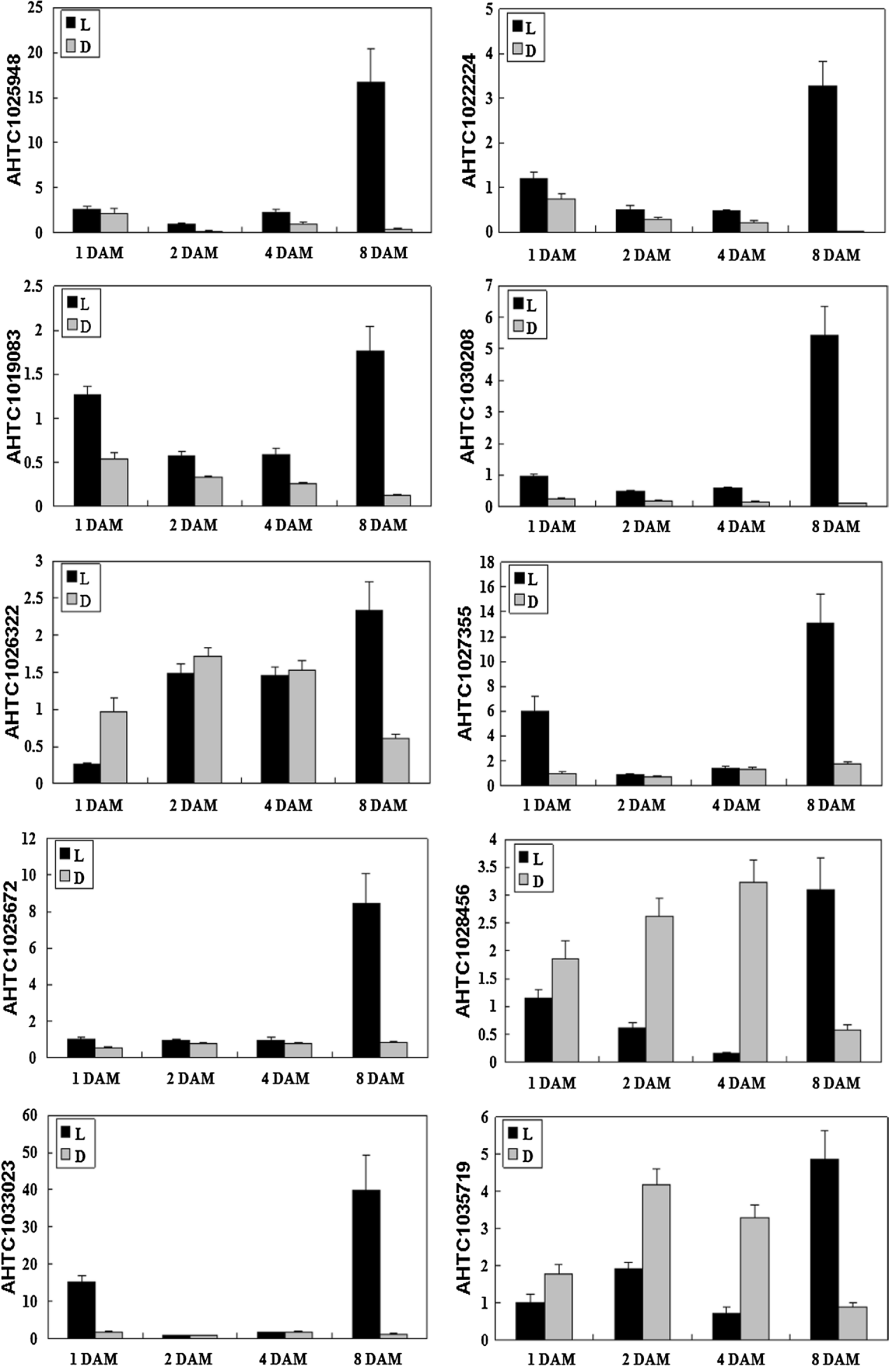
Real time RT-PCR analysis on mRNA transcription of the selected differentially expressed genes. L: aerial pods; D: subterranean pods; 1–8 DAM: the development days of aerial and subterranean pods after marked

In order to confirm the microarrays results, ten genes were randomly selected from seed abortion relative genes based on the GO analysis and subjected to real-time RT-PCR analysis. These genes (as shown in Table 2) were involved in hormone response or cell apoptosis. Primers for these genes were listed in Supplemental Table 5, and the *actin* gene was used as an internal control. As shown in Fig. 6, the expression pattern of 10 selected DEGs analyzed by real-time RT-PCR were consistent with their respective microarrays data. Compared with subterranean pods, all the selected genes were significantly up-regulated in aerial pods at 8 DAM, suggesting that they might function in seed abortion during aerial pods development. In 3 selected genes (AHTC1026322, AHTC1028456, AHTC1035719), their expression levels were higher in subterranean pods at 1–4 DAM, while rapidly decreased on 8 DAM. The expression levels of other 7 selected genes were similarly with each other at 1 DAM to 4 DAM, while significantly up-regulated in aerial pods at 8 DAM.

**Table 2:** The selected differentially expressed genes for real time RT-PCR analysis

Gene ID	Uniprot no.	Gene function	Protein name	Species	E value
AHTC1025948	Q0WQQ1	Hormone response	ADP-ribosylation factor GTPase-activating protein AGD15	*Arabidopsis thaliana*	7.00E−10
AHTC1022224	P33081	Hormone response	Auxin-induced protein 15A	*Glycine max*	5.00E−21
AHTC1019083	P33079	Hormone response	Auxin-induced protein 10A5	*Glycine max*	4.00E−31
AHTC1030208	P33080	Hormone response	Auxin-induced protein X10A	*Glycine max*	8.00E−34
AHTC1026322	Q2HSV9	Hormone response	Transcriptional factor B3; Auxin response factor	*Medicago truncatula*	1.00E−48
AHTC1027355	Q2HVE0	Apoptosis	Leucine-rich repeat	*Medicago truncatula*	2.00E−17
AHTC1025672	Q2YE88	Apoptosis	NB-LRR type disease resistance protein Rps1-k-1	*Glycine max*	5.00E−15
AHTC1028456	Q84ZU8	Apoptosis	R 10 protein	*Glycine max*	3.00E−53
AHTC1033023	Q84ZU5	Apoptosis	R 8 protein	*Glycine max*	8.00E−31
AHTC1035719	Q8W2C0	Apoptosis	candidate resistance protein KR1	*Glycine max*	4.00E−21

Ten DEGs were randomly selected from seed abortion candidate genes to validate the microarrays data by real-time RT-PCR analysis. Based on GO functional categories, they were involved in the biological process of hormone response and cell apoptosis. Gene ID is provided on the left side of the table. Their hits of Uniprot accession number, gene function, protein name, species and E-value are shown in the table

### Measurement of endogenous plant hormone

**Fig. 7:**
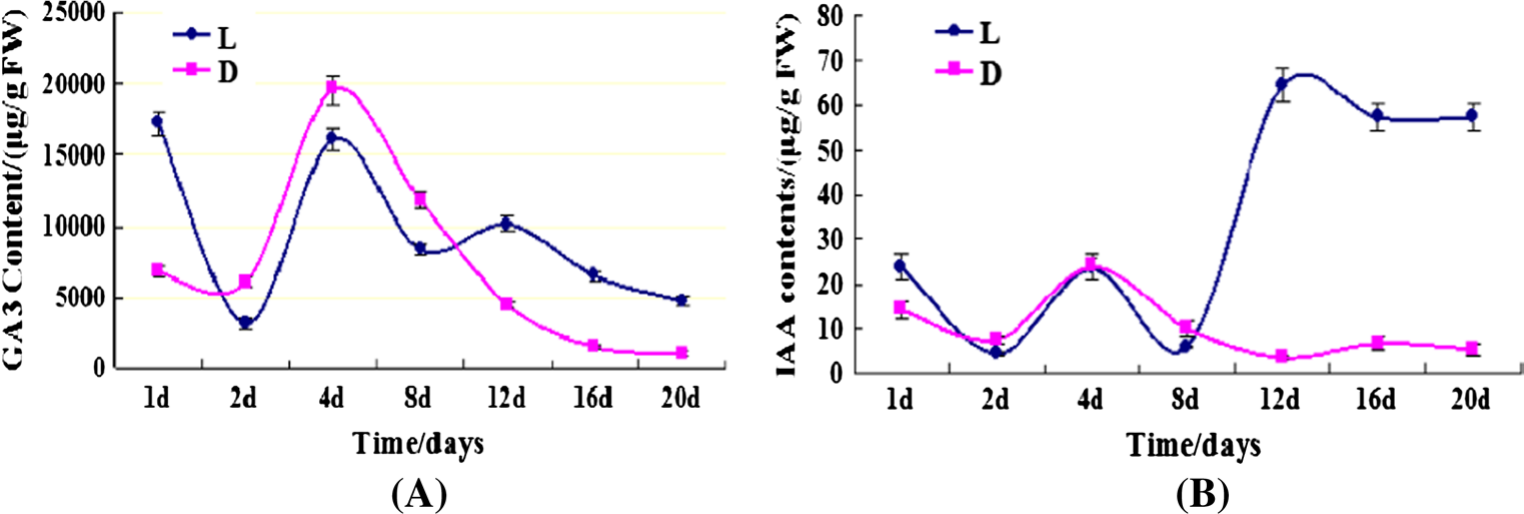
Change of GA_3_ (**a**) and IAA (**b**) content in peanut aerial and subterranean pods development at different days after marked. L: aerial pods; D: subterranean pods; 1–20 days: at the development days after marked. *FW* fresh weight. *Vertical bars* represent standard error of means

With the significant up-regulation of hormone response relative genes in aerial pods, we examined the changes of plant hormone in order to clarify its possible roles in aerial and subterranean pods development. Levels of GA_3_ and IAA were investigated during the whole process by HPLC (Fig. 7). The results showed that in the development from 2 DAM to 8 DAM, the GA_3_ content in subterranean pods was higher than aerial pods, while lower in the other development days. The IAA contents of aerial pods were similarly with each other at 1 DAM to 8 DAM, while significantly increased with six times more than subterranean pods during the development from 12 DAM to 20 DAM. These results well agreed with the microarrays data and real time RT-PCR result of hormone response relative genes, indicating that GA_3_ and IAA might be involved in seed abortion of aerial pods.

## Discussions

In recent years, the powerful tool of microarrays was broadly applied to investigate fruit development, as reported for strawberry (Aharoni and O’Connell 2002), tomato (Alba et al. 2005), pear (Fonseca et al. 2004), and apple (Lee et al. 2007). In this study, we identified 6,203 differentially expressed genes via microarrays analysis, and also detected 4,732 stage-specific expressed genes and 2,401 specific expressed genes only in aerial or subterranean pods across various stages. Additionally, through Gene Ontology analysis, many differentially expressed genes participated in the biological process of cell apoptosis, hormone response, embryonic development and light signaling, which were identified as potential candidate genes responsible for the normal development of subterranean pods and also seed abortion in aerial pods. In a manner consistent with significant up-regulation of auxin response relative genes in aerial pods, the changes of plant hormone IAA potentially contributed to seed abortion during aerial pods development, providing new insight that auxin response factors might be involved in seed abortion. Real time RT-PCR analysis also validated the expression alternation of candidate genes occurring at transcriptional level. Interestingly, seed development is a complex process with fascinating characteristic in seed biology, while several distinctive features can make peanut as an excellent model to study seed and pod development, especially for aerial flowering, gravitropic peg elongation and subterranean fructification.

### DEGs involved in UPS and photosynthesis

Comparison of gene expression profiling between the aerial and subterranean pods development is essential for the elucidation of molecular networks in peanut pod development. Combined with previous RNA-Seq and proteomics analysis (Chen et al. 2013; Zhu et al. 2013), this study further facilitated that the differentially expressed genes and proteins involved in ubiquitin proteasome system (UPS) and cell wall modification, might function as candidates to regulate peanut pod development. As shown in Supplemental Table 6, 21 UPS relative genes were identified as DEGs during aerial and subterranean pods development. These genes might play a critical role in pod swelling process to allow room for embryo development. Gene probesets matching to identified proteins indicated a good consistence of expression alternations between protein and mRNA data. Lots of UPS relative genes were up-regulated in subterranean pods during the early development stages, while significantly decreased at the late stages. However, the expression levels of cell wall modification relative genes were up-regulated in aerial pods. In addition, similar to proteins expression, a great number of photosynthesis relative genes were also significantly up-regulated and enriched in aerial green pods. All these well agreed with the previous RNA-seq analysis, validating the significance of the large number of DEGs changes found in this study.

### Potential functions of auxin response genes in seed and pod development

In recent years, the prominent role of auxin signaling in patterning the early embryo was becoming increasingly clear. When auxin was perceived by its receptor, the auxin response factors (ARFs) would be released to exert their function as activators or repressors of transcription (Möller and Weijers 2009). Studies in *Arabidopsis* had led to an understanding of embryo development processes that were controlled by auxin response factors to coordinate several cell specification and pattern formation (Abel and Theologis 1996; Guilfoyle et al. 1998; Jenik and Barton 2005). For instance, many auxin response factors linked auxin signaling to control the seed development, seed size and cotyledons transition of embryogenesis by regulating cell division and organ growth, such as ARF5 (Okushima et al. 2005; Xing et al. 2011), DR5 (Benkova et al. 2003), ARF7 (Harper et al. 2000) and ARF2 (Schruff et al. 2006). Additionally, the PIN family of auxin efflux facilitators such as PIN1, PIN3, PIN4 and PIN7 (Friml et al. 2002a, b, 2003, 2004), were responsible for the dynamic and shifting pattern of auxin accumulation in the embryo by expressing at stage-specific and tissue-specific during embryonic development.

GAs, auxin, ABA, and ethylene have been implicated in regulating the peanut seed development and pod maturation (Jacobs 1951; Ziv and Kahana 1988; Shlamovitz et al. 1995; Moctezuma and Feldman 1996; Ozga and Reinecke 2003). In this study, a number of differentially expressed genes related to hormone response were identified during aerial and subterranean pods development, while in our previous RNA-seq and proteomics study we could not detect significantly up-regulated genes involved in this pathways. Among them, auxin-related genes accounted for 48.7 % of all hormone response relative genes. Together with IAA content was significantly increased during aerial pods development, many auxin response relative genes (Table 1) were identified as candidates to seed abortion, such as auxin response factor, auxin-induced protein, auxin-repressed protein, auxin response factor GTPase activator, auxin efflux carrier and auxin binding protein. Moreover, the expression pattern of most auxin-related genes were up-regulated during aerial pods development. All these revealed that auxin and auxin response genes potentially played a crucial role in peanut seed and pod development.

### Transcriptional regulation of cell apoptosis and embryonic development

Embryonic development was the main biological process that determined the size and ultimate fate of the seed by cell division and enlargement (Ohto et al. 2009). Based on the embryo abortion during aerial pods development, previous studies underlined the importance of three candidate genes such as two senescence associated genes and one late embryogenesis-abundant gene (Chen et al. 2013). In this study, we identified 16 cell apoptosis relative genes and 17 embryonic development relative genes as candidate genes to seed abortion in aerial pod. We also detected two senescence associated genes and seven late embryogenesis-abundant genes which were specially and significantly up-regulated in the aerial pod. Several reports of senescence-associated genes appeared to trigger senescence program preceding death in response to multiple developmental and environmental signals (Quirino et al. 1999; Gepstein et al. 2003; Lim and Nam 2005; Espinoza et al. 2007). Late embryogenesis abundant proteins accumulated late in plant seed development and played crucial roles in varying stressful environmental conditions (Xu et al. 1996; NDong et al. 2002; Hundertmark and Hincha 2008).

### Transcriptional regulation of light signaling pathway

As Thompson et al. (1985) reported, light leaded to the cessation of embryo differentiation during peg elongation phase, and dark stimulated the resumption of embryo development following quiescence in underground phase. Ten differentially expressed genes involved in light signaling pathway were identified in this study. Phytochrome A-associated F-box protein (AHTC1014391) were identified and significantly up-regulated in subterranean pods at late stages. Some studies suggested that phytochrome was localized in tissue-specific of the developing embryo and integument, which might play an important role in the underground phase, but not in the peg elongation phase (Thompson et al. 1992; Moctezuma 2003). The discovery of these genes indicated that regulation of seed and pod development were important in the acclimation to disparate growth conditions, especially under dark conditions.

### New insights into peanut seed and pod development

Seed formation and pod swelling are of two vital important processes of peanut pod development. However, seed and pod development programs in peanut are highly complex and need to be finely controlled and coordinated by the intervention of several cross talks. Based on the data presented in this work and in our previous investigations, we propose a preliminary overview of the important biological processes occurring during peanut pod development, which are in part schematically represented in Supplemental Fig. 1. The plant hormone auxin significantly increases in aerial pods under light conditions, which in turn directly or indirectly activates auxin response factors to trigger the auxin signal transduction. When the signals transduce to UPS or lignin synthesis pathways, the initiation of pod swelling in peg tips will be suppressed. Alternatively, when the signals transduce to cell apoptosis or embryonic development pathways, it may lead to seed abortion. All these pathways would work together, leading to the seed and pod normally developing. In addition, it is clear that the UPS not only plays an essential role in hormone perception and responses, but also contributes to plant cell division and PCD (Dharmasiri and Estelle 2002; Yanagawa et al. 2002; Kim et al. 2003; Santner and Estelle 2010; Kepinski and Leyser 2002), indicating that UPS may act as a dual coordinator between pod swelling and seed abortion.

## Concluding remarks

In conclusion, seed abortion within aerial pods caused by peg penetration failure is a major limitation of seed yield, seriously impacting on peanut production. In this study, we have performed a comparative investigation of transcriptome profile between aerial and subterranean pods development. Simultaneously, together with endogenous IAA significantly increased in aerial pods, many candidate genes to seed abortion were identified, providing new molecular view that auxin response genes potentially played vital roles in seed and pod development. Although development of peanut aerial and subterranean pods have been studied intensely by DNA microarrays combined with previous RNA sequencing and proteomics analysis, many questions still wait to be answered. It is a key gap in our understanding what and how distinct genes in different seed tissue play important roles in cell division, differentiation and morphogenesis during early seed and embryo development. Our researches of identification and characterization of potential candidate genes and proteins can initiate the long way to unravel regulatory networks that program and coordinate the developmental and physiological events occurring. More detailed analysis by reverse genetic approaches is ongoing to further characterize their possible functional roles in seed and pod development.

## Electronic supplementary material

Below is the link to the electronic supplementary material.

Supplementary material 1 (DOC 26 kb)

Supplementary material 2 (XLS 812 kb)

Supplementary material 3 (XLS 1163 kb)

Supplementary material 4 (XLS 9363 kb)

Supplementary material 5 (XLS 147 kb)

Supplementary material 6 (DOC 23 kb)

Supplementary material 7 (DOC 39 kb)

Supplementary material 8 (XLS 15720 kb)

Supplementary material 9 (FASTA_ONELINESEQ 26120 kb)

Supplementary material 10 (FASTA 5785 kb)

## Abbreviations

DAF: Day after flowering
DAM: Days after marked
UPS: Ubiquitin proteasome system
DEGs: Differentially expressed genes
GO: Gene ontology
PCD: Programmed cell death
