# Supplementary figures and images for: Comparative transcriptome analysis of aerial and subterranean pods development provides insights into seed abortion in peanut
Source: Plant Mol Biol. 2014 May 5;85(4):395–409. doi: 10.1007/s11103-014-0193-x (PMC4152868; doi:10.1007/s11103-014-0193-x)

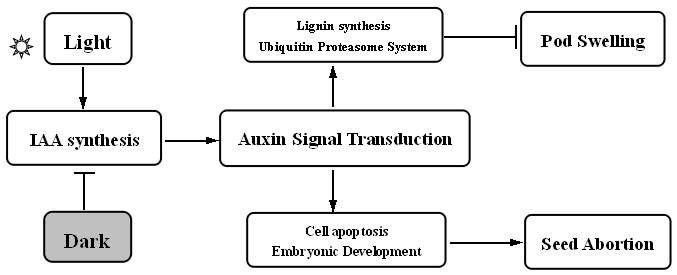


**Supplemental Fig. 1. Overview for auxin regulatory of control in peanut pod development.**

Supplement: Supplementary file 1 — Supplementary material 1 (DOC 26 kb) [file 11103_2014_193_MOESM1_ESM.doc]
