# Supplementary material for: Comparative transcriptome analysis of aerial and subterranean pods development provides insights into seed abortion in peanut
Source: Plant Mol Biol. 2014 May 5;85(4):395–409. doi: 10.1007/s11103-014-0193-x (PMC4152868; doi:10.1007/s11103-014-0193-x)
Supplement: Supplementary file 6 — Supplementary material 6 (DOC 23 kb) [file 11103_2014_193_MOESM6_ESM.doc]

**Supplemental Table 5. Primers used for real time RT-PCR of selected genes in peanut aerial and subterranean pods at different DAM.**

| **Gene ID** | **forward primer (5’-3’)** | **reverse primer (5’-3’)** | **Length (bp)** |
| --- | --- | --- | --- |
| AHTC1025948 | TAGTGGCTTATTTGCGGCTT | TTGGAAGCAGACAAACAGAGAAT | 133 |
| AHTC1022224 | AAGCGAGATGTGATTTGTTGAA | TTTGAGCCAACCTTCATTCC | 126 |
| AHTC1019083 | CTTTCCGCATTCCAGGTATTA | ATCACAAACCGCTTCATCTTATC | 115 |
| AHTC1030208 | GGAAGTCCCGAAAGGCTATCT | GGTCCAAGAAAGCATCCTCACT | 185 |
| AHTC1026322 | TGGAGTGAGAAGGGGCAAC | CCTAAGGGATTTCAATGGACC | 130 |
| AHTC1027355 | AACAATGGAGGGATGGTGG | TCAGGGAAACTAAAGTGAAGAGC | 126 |
| AHTC1025672 | CCTTCATCTCACACCGTTTACC | ACCTCACCTCCCTCCAAGAAT | 135 |
| AHTC1028456 | GTGCGGCATCAGAAAGGTATT | GAAGGAGCCAAAGAGGAGTAGG | 266 |
| AHTC1033023 | TCCTCCTCAAGTTTCCCACG | TGGAGATAGCCAGTGAAACCG | 75 |
| AHTC1035719 | CAGCGTTGTCTGAAGTAGGCA | ACATCCAAGTGGACGGTCG | 143 |
