# Supplementary material for: Comparative transcriptome analysis of aerial and subterranean pods development provides insights into seed abortion in peanut
Source: Plant Mol Biol. 2014 May 5;85(4):395–409. doi: 10.1007/s11103-014-0193-x (PMC4152868; doi:10.1007/s11103-014-0193-x)
Supplement: Supplementary file 7 — Supplementary material 7 (DOC 39 kb) [file 11103_2014_193_MOESM7_ESM.doc]

**Supplemental Table 6. Differentially expressed genes involved in ubiquitin proteasome system during peanut pods development.**

| **Gene ID** | **Uniprot NO.** | **Species** | **Description** | **E-value** |
| --- | --- | --- | --- | --- |
| AHTC1031354 | B9HNA9 | *Populus trichocarpa* | Ubiquitin-activating enzyme E1 | 2.00E-103 |
| AHTC1013032 | B9REZ2 | *Ricinus communis* | Ubiquitin-protein ligase BRE1A | 2.00E-19 |
| AHTC1006293 | B9SVU9 | *Ricinus communis* | Ubiquitin-protein ligase | 1.00E-31 |
| AHTC1018084 | B9SSL4 | *Ricinus communis* | Ubiquitin-protein ligase | 2.00E-116 |
| AHTC1016705 | B9SM61 | *Ricinus communis* | Ubiquitin-conjugating enzyme E2 | 6.00E-35 |
| AHTC1010984 | B9GHK8 | *Populus trichocarpa* | Ubiquitin carboxyl-terminal hydrolase | 1.00E-10 |
| AHTC1000222 | Q06H23 | *Arachis hypogaea* | Ubiquitin carrier protein | 4.00E-84 |
| AHTC1015821 | B9RVT9 | *Ricinus communis* | 26S proteasome regulatory subunit rpn1 | 3.00E-59 |
| AHTC1004998 | B9S397 | *Ricinus communis* | Ubiquitin-protein ligase | 2.00E-14 |
| AHTC1012633 | B9RD12 | *Ricinus communis* | Ubiquitin-protein ligase | 4.00E-16 |
| AHTC1028336 | C5WXW1 | *Sorghum bicolor* | Proteasome subunit alpha type | 5.00E-117 |
| AHTC1006266 | B9T3Z3 | *Ricinus communis* | Ubiquitin-protein ligase | 1.00E-55 |
| AHTC1027562 | A5BYS9 | *Vitis vinifera* | Proteasome subunit alpha type | 5.00E-75 |
| AHTC1018235 | O24240 | *Prunus armeniaca* | Ubiquitin carrier protein | 4.00E-25 |
| AHTC1005410 | B9R9U5 | *Ricinus communis* | Ubiquitin-protein ligase | 5.00E-50 |
| AHTC1001251 | C6SV99 | *Glycine max* | Ubiquitin carrier protein | 1.00E-102 |
| AHTC1006209 | A5CBT2 | *Vitis vinifera* | Ubiquitin carboxyl-terminal hydrolase | 1.00E-25 |
| AHTC1001077 | C6SVE5 | *Glycine max* | Proteasome subunit beta type | 8.00E-123 |
| AHTC1007214 | Q9SPB9 | *Glycine max* | Ubiquitin carrier protein | 1.00E-80 |
| AHTC1003637 | B9T5N2 | *Ricinus communis* | Ubiquitin ligase protein cop1 | 9.00E-167 |
| AHTC1010402 | B9T343 | *Ricinus communis* | Ubiquitin ligase protein cop1 | 7.00E-33 |
